# Efficacy and mechanisms of cannabis oil for alleviating side effects of breast cancer chemotherapy (CBC2): protocol for randomized controlled trial

**DOI:** 10.1186/s12906-024-04426-0

**Published:** 2024-03-23

**Authors:** May Soe Thu, Krit Pongpirul, Mawin Vongsaisuwon, Chanida Vinayanuwattikun, Kamonwan Banchuen, Thunnicha Ondee, Sunchai Payungporn, Phanupong Phutrakool, Preecha Nootim, Pajaree Chariyavilaskul, Sarocha Cherdchom, Kulthanit Wanaratna, Nattiya Hirankarn

**Affiliations:** 1 Joint Chulalongkorn University-University of Liverpool PhD Programme in Biomedical Sciences and Biotechnology, Faculty of Medicine, Chulalongkorn University, Bangkok, Thailand; 2 Department of Microbiology, Faculty of Medicine, Center of Excellence in Immunology and Immune-Mediated Diseases, Chulalongkorn University, Bangkok, Thailand; 3 Department of Infection Biology & Microbiomes, University of Liverpool, Liverpool, UK; 4 Center of Excellence in Preventive and Integrative Medicine, Faculty of Medicine, Chulalongkorn University, Bangkok, Thailand; 5 Bumrungrad International Hospital, Bangkok, Thailand; 6 Department of International Health, Johns Hopkins Bloomberg School of Public Health, Baltimore, MD USA; 7 Department of Surgery, Faculty of Medicine, Chulalongkorn University, Bangkok, Thailand; 8 King Chulalongkorn Memorial Hospital, Bangkok, Thailand; 9 Department of Medicine, Faculty of Medicine, Division of Medical Oncology, Chulalongkorn University, Bangkok, Thailand; 10 Department of Thai Traditional and Alternative Medicine, Ministry of Public Health, Nonthaburi, Thailand; 11 Department of Biochemistry, Faculty of Medicine, Center of Excellence in Systems Microbiology,, Chulalongkorn University, Bangkok, 10330 Thailand; 12 Chula Data Management Center, Faculty of Medicine, Chulalongkorn University, Bangkok, Thailand; 13 Department of Pharmacology, Faculty of Medicine, Chulalongkorn University, Bangkok, Thailand

**Keywords:** Cannabis, Microbiota, Breast Cancer, Quality of Life

## Abstract

**Background:**

In a pilot study using both cannabidiol (CBD) and tetrahydrocannabinol (THC) as single agents in advanced cancer patients undergoing palliative care in Thailand, the doses were generally well tolerated, and the outcome measure of total symptom distress scores showed overall symptom benefit. The current study aims to determine the intensity of the symptoms experienced by breast cancer patients, to explore the microbiome profile, cytokines, and bacterial metabolites before and after the treatment with cannabis oil or no cannabis oil, and to study the pharmacokinetics parameters and pharmacogenetics profile of the doses.

**Methods:**

A randomized, double-blinded, placebo-controlled trial will be conducted on the breast cancer cases who were diagnosed with breast cancer and currently receiving chemotherapy at King Chulalongkorn Memorial Hospital (KCMH), Bangkok, Thailand. Block randomization will be used to allocate the patients into three groups: Ganja Oil (THC 2 mg/ml; THC 0.08 mg/drop, and CBD 0.02 mg/drop), Metta Osot (THC 81 mg/ml; THC 3 mg/drop), and placebo oil. The Edmonton Symptom Assessment System (ESAS), Food Frequency Questionnaires (FFQ), microbiome profile, cytokines, and bacterial metabolites will be assessed before and after the interventions, along with pharmacokinetic and pharmacogenetic profile of the treatment during the intervention.

**Trial registration:**

TCTR20220809001.

## Introduction

Breast cancer patients reported lower quality of life (QoL) globally [1–3], and chemotherapy was a factor in worsening QoL among the patients [4]. Despite standard medical treatment, the impact of integrating complementary and alternative medicine was introduced into the current practice [5]. Therefore, cannabinoids from *Cannabis sativa* have become attractive due to their major impact in reducing pain associated with chemotherapy and tumor-associated symptoms [6–8].

### Clinical evidence of THC and CBD in cancer patients

In a pilot study using both cannabidiol (CBD) and tetrahydrocannabinol (THC) as single agents in advanced cancer patients undergoing palliative care, the doses of THC and CBD were generally well tolerated, and the outcome measure of total symptom distress scores (TSDSs) showed overall symptom benefit [9].

A first nationwide prospective observational cohort had been conducted at 22 sites in 18 provinces of 13 regions in Thailand (Thai Clinical Trial Registry No. TCTR20191231001; Ethical Approval No.36/2562). The study found that practice patterns of Thai cannabis oil could vary across institutions. One drop daily before bedtime was the most common practice and could significantly improve the QoL assessed by using both EQ-5D-5 L and Edmonton Symptom Assessment Scale (ESAS) of cancer individuals including breast cancer.

### Gut microbiota in breast cancer

The human microbiota plays a crucial role in regulating steroid hormone metabolism, primarily through enzymes like hydroxysteroid dehydrogenase [10, 11]. While there’s no conclusive evidence linking dysbiosis to breast cancer, studies comparing breast samples have shown differences in microbial abundance and diversity between healthy individuals and patients [12, 13]. Additionally, less fungal diversity and richness has been seen in cancer patients [14]. Varied hormonal subtypes of breast cancer are associated with distinct fungal signatures in the tumor microenvironment, with estrogen receptor-positive breast cancer patients displaying higher fungal diversity than those with triple-negative breast cancer [15, 16]. Gut dysbiosis can also dysregulate immune responses and their metabolic products like short chain fatty acids influence mucosal immunity [17].

### Skin microbiota in breast cancer

Although the function of breast skin microbiota remains unclear, it is associated with high abundances of skin commensals, notably various *Staphylococcus* species [18]. Potential pathways for microbial transfer to underlying tissue include retrograde transfer through ductal networks, epidermal barrier permeabilization, and migration through nipple-aspirate fluid [18].

### Oral microbiota in breast cancer

There are indications of a relationship between breast cancer and the oral microbiome, with studies suggesting that women with periodontal disease, caused by specific bacteria including the red complex (*Porphyromonas gingivalis, Tannerella forsythia*, and *Treponema denticola*) and the orange complex (*Fusobacterium nucleatum, Prevotella intermedia, Prevotella nigrescens, Peptostreptococcus micros, Streptococcus constellatus, Eubacterium nodatum, Campylobacter showae, Campylobacter gracilis*, and *Campylobacter rectus*), have an elevated risk of breast cancer [19–21].

### Cannabinoids and microbiota

Nearly all the effects of cannabinoids are mediated by cannabinoid (CB) receptors such as CB1 and CB2 receptors, which are predominantly expressed in the brain and immune cells, respectively [22–24]. The gut-brain axis and bacterial metabolites and products have recently come to light as a mechanism by which intestinal bacteria can influence the physiology and inflammation of the central nervous system [25]. These mechanisms are dysregulated and then linked to altered blood-brain barrier permeability and neuroinflammation during dysbiosis [25]. The positive effects of cannabinoids on the GI and immunological systems are lowering intestinal permeability, controlling intestinal bacteria, and reducing inflammation, according to earlier preclinical studies [26, 27].

### Clinical practice in Thailand

Currently, the Herb and Thai Traditional Medicine Development Division, Department of Thai Traditional and Alternative Medicine (DTAM), Ministry of Public Health, Thailand, manufactures Metta Osot (THC 81 mg/ml; THC 3 mg/drop) and Ganja Oil (THC 2 mg/ml; THC 0.08 mg/drop and CBD 0.02 mg/drop), both of which are produced under license number 13/2562 and has been added to the National List of Essential Medicines [28]. The preliminary analysis of the Thai Cannabis Practice Patterns and QoL (Thai Cannabis PQ) study assessed the pain symptoms using the ESAS and QoL using the EuroQoL Group’s 5-dimension, 5-level (EQ-5D-5 L). It revealed that QoL and all ESAS symptoms improved significantly at 1 month (*p* < 0.001), of which feeling of wellbeing (1.00 ± 2.34), pain (0.95 ± 2.39), tiredness (0.93 ± 2.51), and anxiety (0.85 ± 2.25) showed the largest mean differences [29].

### Pharmacokinetic and pharmacogenetic profile

The pharmacokinetic profile of cannabinoids depends on routes of medication such as oral dosage and inhalation. A study revealed that the median value of the time taken to achieve the highest levels (T_max_) of sublingual drops for CBD only and a combination of CBD and THC was 2.17 h (range 1–4 h) and 1.67 h (range 1–3 h) and the highest concentration of the drops (C_max_) was 2.05 ± 0.92 and 2.58 ± 0.68 nanograms/ml, respectively [30]. In contrast, our understanding of the therapeutic potential of cannabinoids remains limited. To enhance our knowledge regarding the proteins and molecules responsible for the transport, action, and metabolism of cannabinoids in humans, we will evaluate specific candidate genes identified in previous reviews [31].

### Adverse events and the safety measures

It is crucial to thoroughly describe the evidence of health effects and hazards in conjunction with the legalization of cannabis use. A summary review of marijuana use revealed that the link between testicular cancer and the drug has the highest quality score, followed by the links to psychosis, suicide deaths, functional and structural integrity, memory and learning, anhedonia, the amygdala, attention, memory, overall activity, and other things [29]. A systematic review of CBD’s side effects found that pneumonia (OR 5.37, 95% CI: 1.17–24.65), decreased appetite (OR 3.56, 95% CI: 1.94–6.53), diarrhea (OR 2.61, 95% CI: 1.46–4.67), somnolence (OR 2.23, 95% CI: 1.07–4.64), and sedation (OR 4.21, 95% CI: 1.18–15.01) were all associated with a higher risk of experiencing [30]. In the current study, the side effects of the cannabis oil have been monitored in the previous clinical practice [29]. The patients suspected of having the cannabis allergy will be excluded during the recruitment, and the adverse events will be assessed using the case record form in every follow up visits.

### Summary of the study

Hence, the current randomized study aims to explore the efficacy of Thai cannabis oil products on the QoL of individuals diagnosed with breast cancer and treated with chemotherapies, along with the study of microbial alteration and diversity by cannabis and pharmacokinetic measures among those patients. We aim to determine the intensity of the pain and QoL experienced by cancer patients using the ESAS and the EQ-5D-5 L forms, respectively, to explore the microbiome profile, cytokines, and bacterial metabolites in hormone-dependent and independent breast cancer patients before and after the treatment with the cannabis oil or no cannabis, and to study the pharmacokinetics parameters and pharmacogenetics profile.

## Materials and methods

### Target population

We include 90 adult breast cancer women patients who were diagnosed with breast cancer or currently receiving chemotherapy at King Chulalongkorn Memorial Hospital (KCMH) with an anticipated starting recruitment approximately in August 2023. The sample size was calculated using the mean ESAS values of the previous study [29].

#### Inclusion criteria

1. Women aged 18–70 years old who were diagnosed with breast cancer
2. Any stages of breast cancer patients who will start or are currently receiving different chemotherapy regimens
3. Breast cancer patients at KCMH during the study period

#### Exclusion criteria

1. Women who have contraindications for chemotherapy, such as anaphylaxis
2. Women who have serious chemotherapy complications such as anaphylactic shock, bone marrow suppression, liver failure, acute kidney injury, and cardiac arrest
3. Women who are scheduled for elective surgery or other procedures requiring general anesthesia during the study
4. Women who are pregnant or lactating and who are planning for pregnancy during the study
5. Women who are terminally ill or inappropriate for placebo medication
6. Women with any other significant disease or disorder which, in the opinion of the investigator, may have put the patient at risk because of participation in the study, or may have influenced the result of the study, or the patient’s ability to participate in the study
7. Women with a known history of substance abuse
8. Women with a history of cannabis ingredients allergy or suspected of having an adverse reaction to cannabis
9. Women using antibiotics within the past 3 months or using NSAID drugs
10. Women consuming probiotic or prebiotic supplements

### Trial design and follow-up

#### Trial site

The prospective randomized placebo-controlled study will recruit the participants at the KCMH in Bangkok, Thailand in 2024.

#### Patient data confidentiality

Only the patients providing written informed consent will proceed to participate in the trial. All the data will be maintained confidentially by the research team.

#### Patient enrollment and recruitment

Patient demographic information will be reviewed by physicians using KCMH’s database. Online (e.g., on Facebook) and onsite poster (e.g., at KCMH) will be used to advertise the study. Patients will be recruited using the inclusion and exclusion criteria. The medical record database will be used to collect clinical and laboratory investigation data related to hormonal breast cancer subtypes, baseline symptoms, current medication including chemotherapy regime and other herbal use, emetic condition, history of surgery, history of other cancers, and history of radiotherapy.

For the recruitment, we will give the trial information to potential participants using surgeons and nurses in each surgical ward or outpatient clinic and a participant information sheet to confirm the participant’s understanding of the trial objective, procedure, benefits, and risks. If potential participants are willing to participate in the trial, they will be asked to sign an informed consent form with the understanding that they can quit the test at any time without any consequence.

#### Patient allocation and blinding

The recruited patients will be randomly allocated into 3 groups: Ganja Oil (THC 1.701 mg/mL or 0.068 mg/drop; CBD 0.003 mg/mL or 0.0001 mg/drop; CBN 0.170 mg/mL or 0.007 mg/drop; COA Ganja Oil TRCM65/05232), Metta Osot (THC 77.622 mg/mL or 3.105 mg/drop; CBD 6.737 mg/mL or 0.2695 mg/drop; CBN 7.406 mg/mL or 0.296 mg/drop; COA Metta Osot TRCM64/33,743 Rev.1), and placebo oil (THC 0 mg/mL or 0 mg/drop; CBD 0 mg/mL or 0 mg/drop; CBN 0 mg/mL or 0 mg/drop; COA Placebo TRCM64/33,742) groups. The allocation will be conducted with a 1:1:1 ratio using block randomization with a block of six by the table of random numbers for creating randomization ID. The research team will provide the randomization code in an opaque envelope to conceal allocation, mask clinicians, data collectors, and patients. Following the cannabis practice patterns of the preliminary study [29], all the participants will be received cannabis or placebo oil once per day before bedtime for 12 weeks.

#### Visits and follow-ups

There will be four visits for the recruited participants during the trial. At the first visit, research investigators will perform the enrollment by checking the eligibility screening, informed consent form, and allocation plan. Then, the blood, saliva, stool, and skin samples will be collected as a baseline and start the treatment as day 1. The next visits will be to assess the adverse drug reaction by a medical officer. At week 6, the blood samples will be collected for assessing the pharmacokinetic parameters. Week-12 visit will be the last visit at which all the participants will be assessed for any adverse reaction, and the blood, saliva, stool, and skin samples will be collected as post-treatment samples.

After the trial, patients will be asked to stop taking cannabis oil from any sources. They will be monitored for another month for withdrawal symptoms.

#### Trial timepoints

Assessment of adverse drug effects will be collected with 4 time points: 0th, 1st, 6th, 12nd weeks of the trial day. The pain symptoms, QoL and diet pattern will be recorded with 3 time points: 0th, 6th, 12nd weeks. The blood collection (3 mL) for pharmacokinetic and pharmacogenetic tests will be performed on 2 time points: 0th, 6th weeks. The blood (10 mL), feces, skin scrapping, and saliva samples will be taken for biochemistry tests (plasma/ serum), microbiome (gut/ skin/ saliva), cytokines (plasma) and bacterial metabolites (serum/ feces) will be analyzed at 2 time points: 0th, 12nd weeks. The overview of the tests and visits of the clinical trial was shown on the following Table 1.

**Table 1:** SPIRIT schedule of enrolment and assessments

#	Timepoint	Week-0 baseline	Week-1 follow-up	Week-6 follow-up	Week-12 follow-up
		T1	T2	T3	T4
1	**Enrolment**				
	Eligibility screening	x			
	Informed consent	x			
	Allocation	x			
2	**Assessment**				
	CRF01 Case Record Form^a^	x			
	CRF02 FW & ADR^b^	x	x	x	x
	CRF03 ESAS	x		x	x
	CRF04 EQ-5D-5 L	x		x	x
	CRF05 Food Frequency Questionnaire	x		x	x
3	**Blood Samples, Serum**				
	Blood Chemistry^c^	x			x
	Cytokines^d^	x			x
	Bacterial Metabolites^e^	x			x
	Pharmacokinetics parameters	x		x	
	Pharmacogenetics parameters	x		x	
4	**Feces Samples**				
	Fecal Microbiome Profile	x			x
	Fecal Bile Acid Profile^f^	x			x
	Fecal SCFAs^g^ and BCFAs^h^	x			x
5	**Skin Samples**				
	Skin Microbiome Profile	x			x
6	**Saliva Samples**				
	Oral Microbiome Profile	x			x

^a^Demographic and Epidemiological Factors: age, weight, height, waist circumference, hip circumference, neck circumference, alcohol consumption, smoking status, occupation, monthly income, method of health payment, and highest education

^b^Follow up and adverse drug reaction: vital signs, physical examination, lab results to assess liver, kidney and heart function, pharmacokinetic record form, drug tolerance assessment form, and adverse event assessment form

^c^Blood Chemistry: lipid profile, liver function test (LFT)

^d^Cytokines: interleukin-17, interferon-gamma, Interleukin-10, tumor growth factor-beta

^e^Bacterial Metabolites: lipopolysaccharide binding protein, sCD14 levels, equol, cadaverine

^f^Fecal Bile Acid Profile: chenodeoxycholic acid, deoxycholic acid, lithocholic acid

^g^SCFAs: butyric acid, propionic acid, acetic acid

^h^BCFAs: iso-butyric acid

### Primary outcome measures

The pain level of each patient will be assessed using the ESAS form at the last visit as primary outcome.

### Secondary outcome measures

#### Pain level assessment

The ESAS at 0^th^, 6^th^ weeks visits will be taken as secondary outcome measures and will be evaluated all the symptoms.

#### Assessment for quality of life

The QoL will be collected at 0th, 6th, and 12nd weeks. At each dimension, it has the coefficients of the level of severity utilized for calculating the utility score in accordance with the program of Health Intervention and Technology Assessment Program (HITAP).

#### Follow up for adverse events

Adverse reactions will be examined using the IRB-approved case report form for adverse drug reaction. The severity of the effects will be evaluated using 5-grade system, and the relationship to the study drugs by 4 statuses.

#### Biochemical assay

Lipid profile and liver function tests will be measured using the biochemistry analyzer and the quantitative analysis will be conducted as a baseline characteristics.

#### Quantitative analysis of cytokines and bacterial metabolites

We will assess alterations in various cytokines, bacterial metabolites, and the microbiome. Quantitative measurement of IL-10 and TGF-β as anti-inflammatory cytokines, and IL-17 and IFN-γ as inflammatory cytokines will be conducted with Flow Cytometry analyzer using human essential immune response panel (13-plex). Bacterial markers such as LBP, sCD14, and equol will be detected using ELISA techniques. Bacterial metabolite concentrations, including short-chain fatty acids, bile acids, and cadaverine, will be determined via HPLC. Then, the expression levels of these parameters before and after treatment will be compared to evaluate the efficacy of the treatment.

#### Microbial abundance and diversity

For the gut, skin, and oral microbiomes, we will compare microbial abundance at the baseline and end of the trial across different treatment arms to investigate how cannabis oil treatment affects pain assessment. The alpha and beta diversity of the microbiota will be analyzed.

#### Determination of pharmacokinetic and pharmacogenetic parameters

The kinetic concentrations of THC and CBD will be quantitatively measured in two visits to determine the pharmacokinetic and the candidate genes for pharmacogenetic parameters.

### Safety

It has been confirmed the effectiveness of the cannabis products in the preliminary analysis and monitored the side effect at the trials.

### Statistical considerations

#### Sample size estimation

We target 30 patients in each arm, considering the maximum follow-up loss of 50%. Since the target population is metastatic breast cancer cases, questionnaires and biological samples such as blood, stool, saliva, and breast skin scrapping will be collected before and after the treatment, which could increase the withdrawal rate at any time during the trial.

#### Statistical analysis

The data collected from different questionnaires, including case report forms, will be prepared in Microsoft Excel, followed by data cleaning and extraction. The statistical analysis will be performed on an intention-to-treat basis using Stata/MP software version 16.0 (StataCorp 2017, College Station, TX). Descriptive statistics like pair t-tests for before and after-treatment groups will be carried out using mean with standard deviation for normally distributed continuous data and median with interquartile range for non-normal distributed continuous data. Categorical data will be presented as counts and percentages using the Chi-square test. Continuous data will be assessed for normal distribution using a histogram and Shapiro–Wilk test. One-way ANOVA with Bonferroni correction will be used for normally distributed continuous data, and the Kruskal-Wallis test will be used for non-normal distributed continuous data among 3 arms.

To identify the alpha diversity, Shannon’s diversity index is used for evenness and Chao 1 index for the richness of operational taxonomic units (OTUs). The Jaccard dissimilarity index is used to detect beta diversity, and it is statistically described by permutational multivariate analysis of variance using distance matrices (PERMANOVA) test. A statistically significant level is defined as *p* < 0.05. Correction for multiple analyses was not done, but they will not be included in the conclusion of the study as factual findings. Sensitivity analysis using some subgroup to show robustness will be considered if the primary outcome is statistical significance.

## Discussion

Thailand actively launched cannabis oil in the market in 2018, subsequently, the FDA (Food and Drug Administration) authorized all hospitals running under the Public Health Ministry for medical cannabis to be available on prescription to patients for approved conditions like cancer chemotherapy for relief of pain, to counter inflammation, and so on [32]. The previous clinical trial as nationwide has been approved the effectiveness of the cannabis oil in cancer patients. The current study is a continual clinical trial to obtain additional information and it is expected to improve the chemotherapy-related symptoms, their QoL, and the adverse effects in breast cancer patients.

As some studies have observed distinct gut bacterial and fungal profiles in various breast cancer subtypes [15, 16], our anticipation is to identify a comparable microbial abundance pattern in both hormone-dependent and independent breast cancer patients. Additionally, we aim to detect alterations in these patterns before and after treatment with cannabis oil and placebo oil, with a focus on pain scores. A noteworthy aspect of this study is its comprehensive examination of the microbiota, encompassing the gut, skin, and oral microbiomes in breast cancer patients.

An advantageous aspects of this study are the effective observation of the immunomodulatory role of the microbiota measuring interleukin-17 (IL-17) and interferon-γ (IFN-γ), interleukin-10 (IL-10) and tumor growth factor-β (TGF-β) and the assessment of the alterations at both the baseline and post-treatment stages or within each treatment group. This approach is expected to yield specific insights into cytokine expression in breast cancer patients and reveal improvement following treatment.

The levels of Lipopolysaccharide-binding protein (LBP) and soluble CD14 (sCD14) serve as markers for bacterial translocation, indicating the potential existence of a gut-breast cancer microbiota axis [33]. It is essential to assess the influence of cannabis oil on preventing the bloodstream transmission of oncogenic bacteria through this axis. Additionally, equol, a potent estrogenic metabolite produced by intestinal bacteria [34], Is of interest. The study also aims to establish a distinct profile of bacterial markers in breast cancer patients and gain insights into their characteristics following treatment.

Short-chain fatty acids (SCFAs), such as acetate, propionate, and butyrate, are present in the intestine exceeding the concentration of 100 mM, and they are mainly produced by 2 major gut bacterial phyla: *Bacteroidetes* and *Firmicutes* [35]. And branched chain iso-SCFAs, which play a role in membrane permeability and fluidity [36], will be investigated as bioactive bacterial metabolites in breast cancer. The SCFAs (beneficial) and iso-SCFAs (potentially harmful) will be indicators for microbial dysbiosis and bowel health [37] in participants, pre- and post-cannabis oil treatment. Primary and secondary bile acids will be diagnosed as bioactive bacterial metabolites in breast cancer. From a range of bile acid profile, deoxycholic acid (DCA) and chenodeoxycholic acid (CDCA) at the physiological level leads to significant reductions in cell invasion, migration, adhesion, and survival but not to cytotoxicity or apoptosis [38]. In addition, lithocholic acid (LCA) stimulates oxidative stress in breast cancer patients, which can reduce cancer cell proliferation [39]. Serum cadaverine level is expected to be differentially expressed at the post-treatment stage since it is related to the breast cancer aggressiveness. Focusing on these bacterial metabolites, we anticipate identifying unique expression levels and substantial improvements through breast cancer treatment.

In addition, we expect to understand the kinetic changes of cannabis and identify the genes associated with variations that contribute to both the therapeutic effects and side effects of cannabis oil.

## Conclusions

The clinical trial will provide the efficacy of Thai cannabis oils in breast cancer female patients in Thailand, mainly in alleviating chemotherapy-related side effects. Furthermore, it will obtain secondary factors such as QoL indexes, alteration of microbiome profile, bacterial metabolites, and cytokines before and after using the cannabis oil, pharmacokinetic parameters, and pharmacogenetic profiles.

## Data Availability

All data produced in the present work are contained in the manuscript.
